# In Vitro Evaluation of Novel Furo[3,2-*c*]coumarins as Cholinesterases and Monoamine Oxidases Inhibitors

**DOI:** 10.3390/molecules30081830

**Published:** 2025-04-18

**Authors:** Mariagrazia Rullo, Alice Benzi, Lara Bianchi, Massimo Maccagno, Guglielmo Marcantoni Taddei, Daniela Valeria Miniero, Giuseppe Felice Mangiatordi, Giovanni Lentini, Leonardo Pisani, Giovanni Petrillo, Cinzia Tavani

**Affiliations:** 1 Department of Pharmacy—Pharmaceutical Sciences, University of Bari “Aldo Moro”, Via E. Orabona, 4, 70125 Bari, Italy; mariagrazia.rullo@uniba.it (M.R.); giovanni.lentini@uniba.it (G.L.); 2 Department of Chemistry and Industrial Chemistry (DCCI), University of Genova, Via Dodecaneso 31, 16146 Genova, Italylara.bianchi@unige.it (L.B.); giovanni.petrillo@unige.it (G.P.); cinzia.tavani@unige.it (C.T.); 3 Department of Medicine & Surgery, LUM University Giuseppe Degennaro Torre Rossi, 70010 Puglia, Italy; danielavaleria.miniero@uniba.it; 4 Department of Biosciences, Biotechnologies and Environment, University of Bari Aldo Moro, Via E. Orabona 4, 70125 Bari, Italy; 5 CNR, Institute of Crystallography, 70126 Bari, Italy; giuseppefelice.mangiatordi@cnr.it

**Keywords:** furo[3,2-*c*]coumarins, acetylcholinesterase, butyrylcholinesterase, monoamine oxidases, docking simulations, enzyme inhibitors, cytotoxicity

## Abstract

Coumarin represents a privileged structural motif that is quite common in nature-derived and synthetic bioactive molecules. Some of us have recently described the straightforward preparation of complex furo[3,2-*c*]coumarins through a sequential double coupling protocol. Aiming at finding novel chemical probes for the modulation of key anti-Alzheimer’s targets, a small subset of furo[3,2-*c*]coumarin prototypes and their non-aromatic synthetic precursors were tested in vitro as inhibitors of ChEs (acetyl- and butyrylcholinesterase, AChE and BChE) and MAOs (monoamine oxidases A and B, MAO A and MAO B). All compounds were low-micromolar AChE inhibitors devoid of toxic effects against SH-SY5Y cells. Lineweaver-Burk plots and docking simulations suggested mixed-type kinetics for inhibitor **3d** (IC_50_ = 4.1 μM toward AChE). Its promising inhibitory profile encompasses additional, highly selective, activity against monoamine oxidase B, with a submicromolar IC_50_ value (561 nM).

## 1. Introduction

Coumarin (1,2-benzopyrone, or 2*H*-1-benzopyran-2-one according to IUPAC classification) and its derivatives are lactones of *o*-hydroxycinnamic acid widely distributed in nature as free forms or glycosides [1]. More than 1300 structurally diverse coumarins have been identified from different natural sources [2], in particular within the plant kingdom. These oxygen-containing heterocycles can be mainly found in higher plants, where they are produced as secondary metabolites, along the shikimate and the general phenylpropanoid pathways [3], and enable defensive mechanisms. To a lesser extent, coumarins can be isolated from fungi and bacteria. Natural coumarins can be classified into six different subsets according to the core structure: simple coumarins, linear and angular furanocoumarins, linear and angular pyranocoumarins, biscoumarins, benzocoumarins, and coumestans [4].

Coumarin takes its name from coumarou, a vernacular French term used to indicate the bean of the Tonka tree (*Dipteryx odorata*) that was formerly known with the botanical name *Coumaruna odorata* [5]. Isolated by Vogel more than two centuries ago [6], coumarin itself is largely used as fragrance in perfume industry and cosmetic products thanks to its flavoring vanilla-type aroma. The consumption of certain types of cinnamon represents the main source for human exposure to coumarin and its derivatives, that can be found in lower concentrations also in many other edible plants (vegetables, apricots, strawberries, cherries, etc.). Hepatotoxic side effects have been proven in rats and dogs, although coumarin has not been classified as carcinogen by the International Agency for Research on Cancer (IARC).

**Chart 1:**
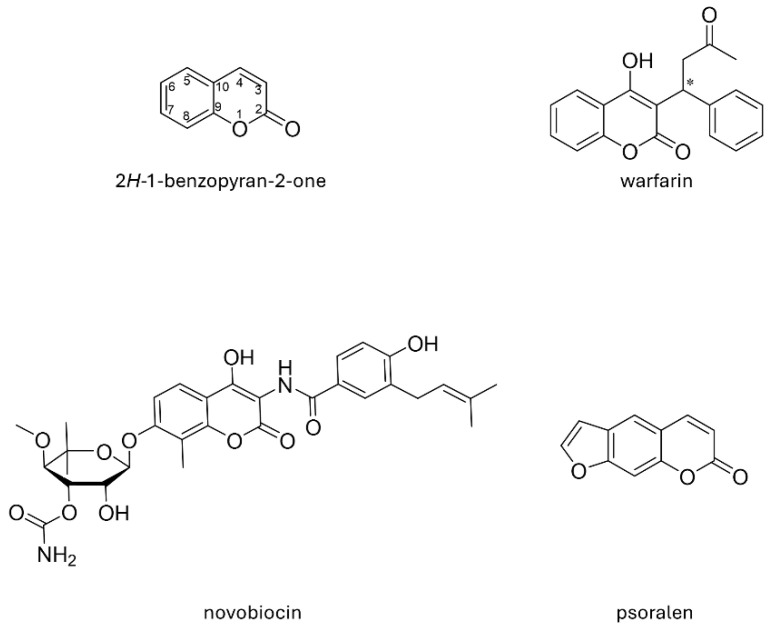
The numbering of 2*H*-1-benzopyran-2-one and some examples of approved drugs containing the heterocyclic core of coumarin.

Although coumarin is not allowed as a food additive by the EU regulation [4], the nature-friendly heterocyclic core of coumarin is present in different classes of FDA approved drugs (Chart 1). Novobiocin and the more complex coumermycin are coumarin-containing topoisomerase inhibitors that act as antibiotics upon targeting bacterial DNA Gyrase B, thus interfering with ATPase activity and blocking ATP-dependent DNA negative supercoiling [7]. 4-Hydroxycoumarins as warfarin, acenocoumarol, and phenprocoumon are vitamin K antagonists used as oral anticoagulants under strict medical monitoring [8]. They prevent the γ-carboxylation reaction in the activation process of clotting factors [9].

From a synthetic viewpoint, the coumarin is an easy-to-handle and chemically tractable fragment, offering many anchoring points for exploring molecular diversity. Thus, over the years, huge efforts have been devoted to optimizing synthetic protocols targeted to suitably modified structures, e.g., by means of introducing different functional groups as substituents all around the heterocyclic core [10,11,12,13]. The presence of six branching positions (3-, 4-, 5-, 6-, 7-, 8-) makes coumarin a building block with outstanding scaffolding properties, devising molecular growing vectors toward all spatial directions [14,15]. These features increase the chances of finding suitable substitution patterns and appropriate geometries for binding interactions with potential drug targets [16]. Indeed, the chemical decoration of the coumarin core has long been exploited to developing bioactive molecules modulating the activity of different proteins (carriers, enzymes, and receptors) such as multidrug-resistance efflux pumps [17], acetylcholinesterase [18,19], STAT3 [20], monoamine oxidases A and B (MAO A and B) [21,22], carbonic anhydrases [23], aromatase [24], heat shock protein 90 [25], serine proteases [26], and many others. More recently, some of us have devoted great attention to the development of dual AChE-MAO B coumarin-based inhibitors as possible anti-Alzheimer’s agents [27,28,29,30].

Another possibility for coumarin core decoration/modification consists of the annulation approach, as also fused coumarins can be found in nature. Among these, linear furanocoumarins with the furane ring fused to the benzene ring of coumarin (psoralen, xanthotoxin, i.e., furo[3,2-*g*]chromenones) are well-studied compounds classified as primary photosensitizing agents that can be activated by UV light (320–380 nm wavelength) and produce phototoxicity through DNA cross-linking or fatty acids aberrations at the membrane phospholipid level. Other types of 3,4-heterocycle-fused coumarins, including angular furocoumarins [31], have been reported in the literature as bioactive compounds [32]. Recently, some of us became interested in novel fused-heteropolycycles characterized by the presence of furan or dihydrofuran rings as potential antioxidants mimiking phenolic and polyphenolic natural compounds [33]. Accordingly, a few classes of furo-fused heterocycles were synthesized by an annulation protocol exploiting, on one side, the bidentate O/C nucleophilic reactivity of phenolic moieties embedded in different heterocycles [34,35], and, on the other side, the bidentate C/C electrophilic character of nitrostilbenes. In particular, the application of the annulation protocol to 4-hydroxycoumarin [35] allowed the access to angular dihydrofuro[3,2-*c*]coumarins which were then subjected to oxidative aromatization to the corresponding furo[3,2-*c*]coumarins, characterized by previously unreported functionalization patterns.

Actually, both natural [36] and synthetic linear furo[3,2-*g*]coumarins [37] have been already described as cholinesterases (acetyl- and butyrylcholinesterase, AChE, and BChE) inhibitors. Anti-AChE activity still represents a key player in Alzheimer’s disease (AD) therapy [38], even if other mechanisms have been evoked as more valuable treatment strategies [39]. Along the so-called multitarget strategy to combat AD [40], the additional inhibition of MAOs has been envisaged as a powerful tool to counteract oxidative stress conditions [41], a common feature of neurodegenerative diseases.

With the aim of improving the portfolio of multitargeting blockers, in this manuscript, we report the in vitro biological evaluation of some angular furo[3,2-*c*]coumarins, made available by the annulation protocol cited above, as human AChE, BChE, MAO A, and MAO B inhibitors. In order to assess the significance (if any) of the spatial molecular arrangement on the inhibition activity, some precursor dihydrofuro[3,2-*c*]coumarins, characterized by a lower extent of coplanarity of the heterocyclic fused system, were also subjected to evaluation. Moreover, docking simulations were run in order to explain the plausible binding modes toward key target enzymes. The cytotoxicity of the whole series was assessed toward human neuroblastoma cells (SH-SY5Y) and is reported herein.

## 2. Results and Discussion

### 2.1. Synthesis of Dihydrofuro[3,2-c]coumarins ***2*** and Furo[3,2-c]coumarins ***3***

**Scheme 1:**
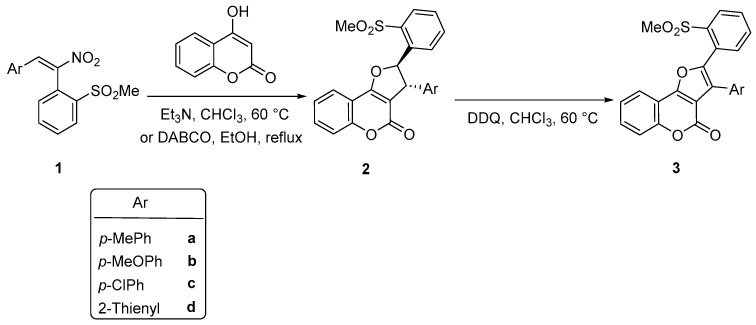
Synthesis of dihydrofuro[3,2-*c*]coumarins **2** and of relevant aromatized furo[3,2-*c*]coumarins **3**.

The dihydro[3,2-*c*]furocoumarins **2** and furo[3,2-*c*]coumarins **3** evaluated herein have been synthesized as recently reported by some of us via the sequence sketched in Scheme 1 [35] from 4-hydroxycoumarin and nitrostilbenes **1** [42], which generates the dihydro derivatives **2** as single (*anti*) diastereomers, then subjected to aromatization.

Experimental details for the synthesis of the model thiophene derivatives **2d** and **3d** are reported in the Experimental section. The syntheses of all the dihydrofuro- and furocoumarins tested herein have been already reported together with full physical and structural characterizations [35]. Compared to literature methods for the synthesis of angular furo[3,2-*c*]coumarins [31,32,43,44,45,46,47,48], the approach of Scheme 1 significantly enriches the available structural diversity as it allows to modulate the geometrical flexibility by means of the saturation degree of the furane ring (e.g., **2** vs. **3**). In this regard, it should be remarked that, from a mechanistic viewpoint, the possibility to isolate the dihydrofurocoumarins **2** heavily rests on the presence of the electronwithdrawing *o*-methylsulfonyl-substituted benzene ring, which limits the negative charge delocalization onto the nitro group along with the initial Michael coupling, thus favoring its elimination upon ring closing to furane **2** [35].

### 2.2. In Vitro Biological Evaluation

All compounds have been tested as inhibitors of ChEs and MAOs from human sources, by applying the well-established spectrophotometric Ellman’s method [18,49]. Compounds **2a**–**d** were assayed as diastereomeric mixtures. Data are reported in Table 1 and indicated that no clear structure-activity relationships (SARs) can be derived. However, some in vitro results are worth noting. In fact, all compounds inhibited *h*AChE at the low micromolar level with IC_50_ values close to each other (4.0 μM < IC_50_ < 6.1 μM). Aromatized derivatives **3a, b, d** were equipotent AChE inhibitors compared to the dihydro analogs **2a, b, d** (**2a** vs. **3a**, **2b** vs. **3b**, **2d** vs. **3d**). Both partially saturated and conjugated coumarins can be considered selective AChE inhibitors, since they exhibited very low percentage of inhibition (**2a**, **2b**, **3b**, **3d**) or the absence of inhibitory activities toward hBChE (**2c**, **2d**, **3a**).

**Figure 1:**
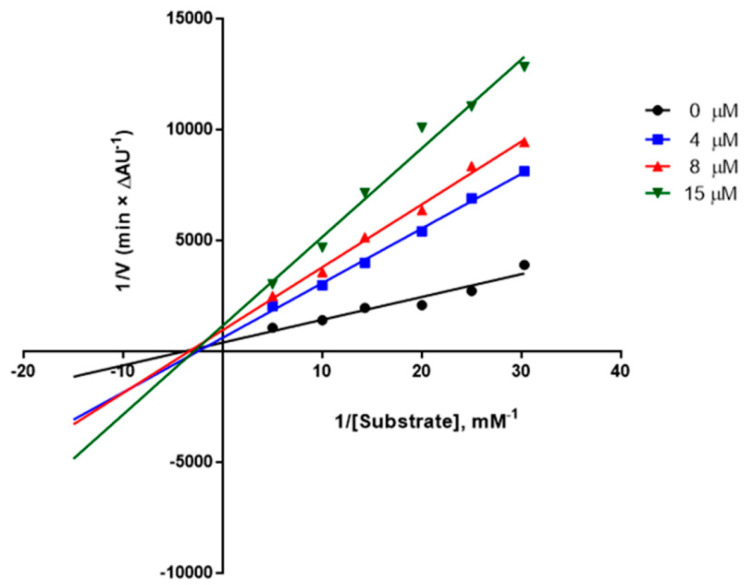
Lineweaver-Burk plots of inhibition kinetics of compound **3d** toward *h*AChE. Reciprocals of enzyme activity (*h*AChE) vs. reciprocals of substrate (*S*-acetylthiocholine) concentration in the presence of different concentrations (0–15 μM, reported with a different color code in insets) of inhibitor.

The most active achiral compound (**3d**, IC_50_ = 4.1 μM toward AChE) was enrolled in the investigation of inhibition kinetics. Lineweaver-Burk plots at different inhibitor concentrations (0–15 μM) in Figure 1 indicated a mixed-type or a non-competitive inhibition mechanism for AChE with a *K*_i_ = 9.7 ± 0.6 μM. This behavior can suggest the possibility for this compound to occupy the peripheral anionic subsite (PAS) of AChE, at least in part. From a three-dimensional viewpoint, the active site of AChE is characterized by the presence of a narrow gorge, about 20 Å long, spanning from the catalytic anionic subsite (CAS), close to the catalytic triad, to the peripheral anionic subsite [50,51]. PAS-binding property can have a beneficial effect against Alzheimer’s, since PAS has been postulated to exert a chaperone-like activity that accelerates amyloid deposition into fibrils [52].

Moreover, this series of compounds was also tested as potential inhibitors of both monoamine oxidase isoforms (MAO A and B). In fact, the blockade of MAO B can endorse the potential as neuroprotective or anti-Alzheimer’s tools [53,54]. As inferred by the inhibition data from Table 1, **2a**, **b** showed comparable activity toward both isoforms, lacking the necessary selectivity toward MAO B that could reduce the risk of sympathomimetic side effects (e.g., cheese reaction [55]). Unfortunately, coumarin **2d** was significantly more active toward MAO A than B.

Surprisingly, the aromatization of the furane ring provided selective, albeit moderate, MAO B inhibitors **3a** and **b**, that were inactive toward the A isoform. It is worth noting that congener **3d** inhibited *h*MAO B activity at the submicromolar level, returning an IC_50_ equal to 0.561 μM. Moreover, this compound was highly selective toward the B isoform, showing no inhibition of MAO A activity upon incubation at 10 μM concentration and deserving further attention as a potential multitarget tool.

### 2.3. Cytotoxicity

**Figure 2:**
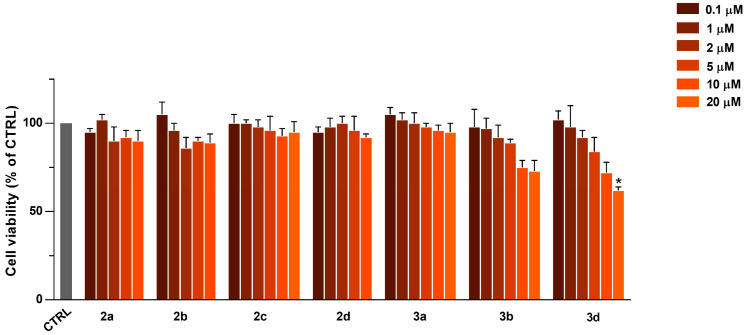
Cell viability of neuroblastoma SH-SY5Y cells incubated with compounds **2a–d** and **3a, b, d** (0.1–10 µM or 0.1–20 µM concentration range, 24 h incubation), as determined by MTT assay. Bars represent the mean ± SD of three independent experiments, each performed in triplicates and referred to untreated control cells (CTRL, 100% value, in the absence of compounds). Statistical significance was calculated using one-way analysis of variance (ANOVA) followed by Dunnett’s multiple comparison test. Levels of significance are referred to untreated cells: * *p* < 0.05.

The cytotoxicity of compounds **2a–d** and **3a, b, d** was assessed upon co-incubation with human neuroblastoma cells and is reported as bar plots in Figure 2. For the diastereomer mixtures of compounds **2a**, **2b**, and **2d**, the highest concentration tested was equal to 10 micromolars because of precipitation in the assay conditions at higher concentrations (20 μM). As can be observed in Figure 2, all compounds showed negligible cytotoxic effects at the studied concentrations, chosen within the same order of magnitude than IC_50_ values toward AChE.

### 2.4. Computational Studies

**Figure 3:**
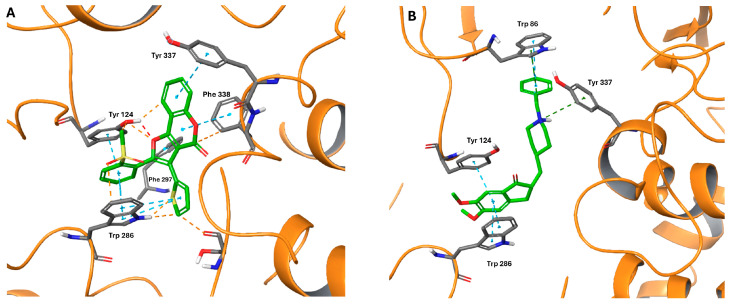
Top-scored binding pose of compound **3d** (**A**) and donepezil (**B**) docked within *m*AChE (PDB 7QAK, docking score = −6.573 kcal/mol and −12.549 kcal/mol, respectively). Ligands and relevant amino acid residues are rendered as sticks, while protein is represented as a cartoon. Colors are in accordance with the atom code, C atoms in green and gray for ligand and amino acid, respectively. Dotted blue lines represent π–π stacking; dotted orange and red lines represent bad and ugly contacts/clashes, respectively [57,58].

Molecular docking simulations were run for the most promising in vitro dual-targeting inhibitor (**3d**) within the binding site of AChE and MAO B to detect plausible binding poses and to better rationalize its biological activity. As for the protein template, high-resolution crystal structure of *mus musculus* AChE (*m*AChE) binary complex with a coumarin-based inhibitor (PDB code 7QAK [56]) was recruited, because of high sequence homology with the human enzyme and ligand similarity. Notably, the predicted binding mode for compound **3d** appears to be primarily driven by π–π stacking interactions. In agreement with the kinetic analysis, inhibitor **3d** settles in the PAS and in the mid-gorge of the target enzyme as illustrated in Figure 3A. The low inhibitory activity can be ascribed to several unfavorable interactions (i.e., steric clashes indicated by dotted orange and red lines in Figure 3) that were detected in the complex for all top-scored binding poses, being the computed docking score −6.573 kcal/mol for the best-ranked one. This value closely matches the in vitro experimental activity scale, hence supporting the reliability of the performed docking study. As a matter of fact, the free-energy calculation returned a much lower value for **3d** than the score obtained upon redocking the coumarin-based native ligand of 7QAK crystallographic complex (docking score = −12.023 kcal/mol), possessing an IC_50_ at the nanomolar level (120 nM) toward *h*AChE [56]. In details, the sulphonyl-substituted phenyl ring of compound **3d** is anchored to the PAS of the target enzyme by a sandwich-like arene-arene interaction directed to the indole side chain of Trp286 (distance between centroids: 3.5 Å) at the entrance of the enzymatic cavity and Tyr124 (distance between centroids: 3.4 Å). A similar interaction geometry was retrieved by donepezil, a standard AChE inhibitor marketed against AD, as shown in Figure 3B illustrating short-range favorable arene-arene interactions arranging the dimethoxyphenyl ring in front of both Trp286 (3.6 Å) and Tyr124 side chain (3.9 Å). However, donepezil behaves as a dual binding-site inhibitor able to fill the cavity from PAS to CAS, indeed it performs key additional interaction stacking parallel to Trp86 at the CAS level. The inhibitory potency of this drug, higher than both **3d** and the native ligand, was further confirmed by the docking score calculated in our simulation (−12.549 kcal/mol). Docking protocols for **3d**, including water molecules from the active site (e.g., 737 and 756), returned a lower docking score (−5.730 kcal/mol), likely due to steric clashes and water displacement by the bulky and hydrophobic ligand (data not shown).

**Figure 4:**
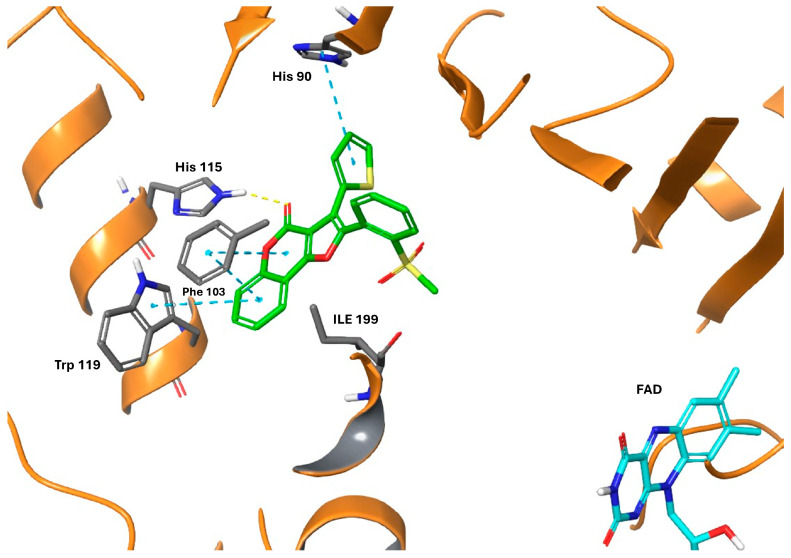
Top-scored binding pose of compound **3d** docked within *h*MAO B (PDB 7P4F, docking score = −12.602 kcal/mol). Ligands and relevant amino acid residues are rendered as sticks, while protein is represented as a cartoon. Colors are in accordance with the atom code, with C atoms in green and gray for ligands and amino acids, respectively. Dotted blue lines represent π−π stacking; dotted yellow lines represent hydrogen bonds [57,58].

Thanks to ligand similarity, the X-ray binary complex coded as 7P4F in PDB [56] between human MAO B and a coumarin inhibitor was enrolled to dock **3d** into this enzyme. Upon applying an induced fit protocol, to ensure target side chain flexibility, a good docking score was obtained (−12.602 kcal/mol) in agreement with the submicromolar potency of **3d** as a MAO B inhibitor. As highlighted in Figure 4, the bulky ligand is accommodated within the entrance cavity and does not fill the substrate cavity gated by Ile199 and Tyr326, leaving the aromatic cage empty. The complex is stabilized by strong arene–arene interactions involving the coumarin motif. In detail, this heterocycle stacks parallel to Phe103 (3.7 Å distance), and the lactone carbonyl is hydrogen bonded with the His115 side chain (N···O distance of 3.2 Å). The thiophene ring of **3d** interacts with His90.

## 3. Materials and Methods

### 3.1. Chemistry

Synthetic and analytic procedures are fully described in the cited ref. [35]. Hereinafter, for the sake of clarity, the experimental details for the synthesis of the model thiophene derivatives **2d** and **3d** are reported.

#### 3.1.1. Reaction of Substrates **1d** with 4-Hydroxycoumarin

In a round-bottom flask, the substrate **1d** (0.16 mmol) was dissolved in ethanol (1.5 mL); then, 4-hydroxycoumarin (0.32 mmol) and 1,4 diazabicyclo[2.2.2]octane (DABCO, 0.24 mmol) were added. The reaction mixture was refluxed under magnetic stirring until the substrate disappeared (TLC). **2d** precipitated as a white solid in EtOH (74%), so it could be separated by filtration, washed with ethanol, and subjected to aromatization with no need for further purification. 

#### 3.1.2. Aromatization of **2d** with DDQ

The dihydrofurocoumarin **2d** (0.12 mmol) was dissolved in chloroform (3.0 mL) and 2,3-dichloro-5,6-dicyano-p-benzoquinone (DDQ, 0.48 mmol) was added. The reaction was heated at 60 °C under magnetic stirring until completion (monitored by TLC). Then, an aqueous saturated NaHCO_3_ solution (8 mL) was added, and the resulting mixture was extracted with CHCl_3_ (3 × 15 mL). The combined organic layers were dried over anhydrous Na_2_SO_4_, filtered, and concentrated in vacuo. The reaction mixture was purified by column chromatography (petroleum ether/ethyl acetate 1:1) to afford the desired **3d** (99%).

### 3.2. In Vitro Enzymatic Inhibition Assays

All human enzymes, substrates and reagents were from Sigma-Aldrich (Milan, Italy). Experiments were run in 96-well plates from Greiner Bio-One (Kremsmenster, Austria) with an Infinite M1000 Pro multiplate reader (Tecan, Cernusco sul Naviglio, Italy). The inhibition of ChEs was determined by following Ellman’s spectrophotometric assay in transparent, flat-bottom plates [18]. For studies addressing MAOs inhibition the spectrofluorimetric protocol was based on the oxidative deamination of kynuramine to 4-hydroxyquinoline measured in black, flat-bottom polystyrene plates [29]. Incubations were performed in triplicate and results were expressed as the mean ± SEM from 3 independent experiments. The values of IC_50_ were calculated by nonlinear regressions using GraphPad Prism 5.00 (GraphPad Software, San Diego, CA, USA). Kinetic studies were performed with the same test conditions, using six concentrations of substrate (from 0.033 to 0.2 mM) and four concentrations of inhibitor (0–15 μM). Apparent inhibition constants and kinetic parameters were calculated within the “Enzyme kinetics” module of Prism.

### 3.3. Molecular Docking Simulations

Docking simulations were performed using the crystal structure of compound **1** in ref [56] in complex with *m*AChE (PDB code 7QAK) and *h*MAO B (PDB code 7P4F) retrieved from the Protein Data Bank. The protein pretreatment to remove water molecules, assign bond orders, add hydrogen atoms, create disulfide bonds, fill in missing side chains and loops using Prime [59] and cap termini was carried out with the Protein Preparation Wizard module available from Schrödinger Release 2024-4. Protonation states at pH 7.0 ± 2.0 and tautomers for histidine residues were predicted with the Epik function and default parameters were used for the optimization of hydrogen-bond assignment. A restrained energy minimization step only on hydrogens was finally applied to the protein using the OPLS4 force field [60]. The ligand preparation was performed using the LigPrep [61] module in the Schrödinger Suite. Compound **3d** (Table 1) was initially drafted in the Maestro [57] panel; then protonation states were calculated at pH 7.0 ± 2.0 retaining specific chiralities and applying the OPLS4 force field. 

As for the docking calculations performed on AChE, the “Receptor Grid Generation” panel of Glide [58] was used to generate the grid files with grid points calculated within an enclosing box of 20 Å defined by the X-ray coordinates of **1** [56] from 7QAK. Docking calculations were performed for each ligand using the Glide module of Schrödinger, using standard (SP) mode and default settings (van der Waals radius scaling parameters: scaling factor of 0.80 and partial charge cut-off of 0.15; dock flexibly; add Epik state penalties to docking score; perform post-docking minimization). 

For docking runs on *h*MAO B, the Induced Fit Docking protocol from Schrödinger Release 2024-4 was employed to ensure a reliable binding pose of compound **3d** within the protein cavity. Specifically, the protocol was applied by centering the grid box on the co-crystallized ligand while maintaining all default parameters.

The scoring function of Glide known as “Docking score” was used for ranking the binding affinity. Pictures were elaborated using Maestro, available from the with Schrödinger Release 2024-4.

### 3.4. Cell-Based Experiments

The cell viability of human neuroblastoma SH-SY5Y cells was measured as previously reported in ref. [29]. To assess the potential cytotoxicity of **2a**–**d**, **3a**,**b**, and **3d**, compounds under study were co-incubated at 0.1–20 µM concentration range (0.1–10 µM for **2a**, **2b**, **2d**) for 24 h. Then, viable cells were determined by MTT [3-(4,5-dimethylthiazol-2-yl)-2,5-diphenyl tetrazolium bromide] assay and results (as percentage) are referred to untreated cells (control, CTRL, cells without compounds, 100% viability). Briefly, cells were plated at a concentration of 4.5 × 10^4^ cells/well and cultured in Dulbecco’s modified Eagle’s medium (DMEM) high glucose supplemented with 10% (*v*/*v*) heat-inactivated fetal bovine serum (FBS), 2 mM L-glutamine, 100 U/mL penicillin, and 100 μg/mL streptomycin at 37 °C in an atmosphere of 5% CO_2_ in 96 wells/plate. At 80% confluence, the medium was removed, and cells in serum-free DMEM were treated with compounds **2a**–**d**, **3a**,**b**, and **3d** at concentrations ranging from 0.1 to 20 µM for 24 h at 37 °C in 5% CO_2_. Then, cells were incubated for 2 h at 37 °C and 5% CO_2_ with 0.5 mg/mL of MTT, and the formazan crystals in the cells were solubilized with absolute ethanol. The amount of the formazan product was determined by absorbance values measured at 545 nm using a multilabel plate counter Victor V3 (Perkin Elmer, Milan-Italy) with a reference wavelength of 690 nm. Cell viability was expressed as a percentage of the negative control (CTRL), represented by untreated cells, which was set at 100%. Data are the mean ± SD from three independent experiments, each performed in triplicate and analyzed using GraphPad Prism 5.0 (GraphPad Software Inc., San Diego, CA, USA). Statistical significance was calculated using a one-way analysis of variance (ANOVA) followed by Dunnett’s multiple comparison test. Levels of significance refer to untreated cells: * *p* < 0.05.

## 4. Conclusions

In this manuscript, we described the preparation and biological screening of novel oxygen-containing heterocycles, bearing a furo[3,2-*c*]coumarin motif. Studied angular coumarins were able to inhibit AChE at a low micromolar level and behaved as non-toxic compounds, showing negligible damage when co-incubated with human neuroblastoma cells. Compound **3d**, carrying a 2-substitued thienyl ring and exhibiting IC_50_ = 4.1 μM toward *h*AChE, underwent further investigations. Its kinetic analysis showed a mixed-type or non-competitive binding mode that was further corroborated by docking simulations. This behavior outlined the possible occupancy of PAS, preventing pro-aggregating features in amyloid fibrilization. Additionally, **3d** displayed a selective inhibition of *h*MAO B (IC_50_ = 0.561 μM). Taken together, these findings represent a piece of work highlighting the successful use of even more complex coumarins, such as angular-fused structures, in the discovery of bioactive molecules. Having in hand a straightforward synthetic protocol, hit optimization will be addressed to deepen structure–activity relationships.

## Figures and Tables

**Table 1:** In vitro inhibition data toward *h*ChEs and *h*MAOs for compounds **2a**–**d** and **3a, b, d**.

Compound	Ar	IC_50_ (μM) or % Inhibition at 10 μM *^a^*			
		*h*AChE *^b^*	*h*BChE *^c^*	*h*MAO A *^d^*	*h*MAO B *^d^*
**2a**	4-MePh	5.5 ± 0.7	27 ± 12%	47 ± 3%	40 ± 8%
**2b**	4-MeOPh	6.1 ± 0.3	29 ± 11%	45 ± 1%	39 ± 6%
**2c**	4-ClPh	4.0 ± 0.7	n.i. *^e^*	16 ± 14%	11 ± 6%
**2d**	2-Thienyl	4.0 ± 0.5	n.i. *^e^*	42 ± 4%	17 ± 10%
**3a**	4-MePh	4.4 ± 0.5	n.i. *^e^*	n.i. *^e^*	32 ± 0%
**3b**	4-MeOPh	5.7 ± 1.0	19 ± 9%	n.i. *^e^*	43 ± 1%
**3d**	2-Thienyl	4.1 ± 0.7	21 ± 3%	n.i. *^e^*	0.561 ± 0.085
**donepezil**		0.017 ± 0.004			
**tacrine**			0.032 ± 0.011		
**clorgyline**				0.020 ± 0.003	
**safinamide**					0.037 ± 0.001

*^a^* Values are the mean of three independent experiments ± SEM. *^b^* Human AChE. *^c^* Human BChE. *^d^* Human recombinant MAO A and B on Supersomes. *^e^* n.i. = no inhibition or inhibition lower than 5% at 10 μM concentration.

## Data Availability

The data presented in this study are available on request from the corresponding authors.
